# Extracellular vesicles as precision therapeutics for psychiatric conditions: targeting interactions among neuronal, glial, and immune networks

**DOI:** 10.3389/fimmu.2025.1454306

**Published:** 2025-04-08

**Authors:** Ivana Kawiková, Václav Špička, James C. K. Lai, Philip W. Askenase, Li Wen, Zdeněk Kejík, Milan Jakubek, Karel Valeš, Filip Španiel

**Affiliations:** ^1^ National Institute of Mental Health, Klecany, Czechia; ^2^ Department of Medicine, Yale School of Medicine, New Haven, CT, United States; ^3^ Department of Biology, Hartford University, West Hartford, CT, United States; ^4^ Institute of Physics of the Czech Academy of Sciences, Prague, Czechia; ^5^ Department of Biomedical and Pharmaceutical Sciences, Idaho State University College of Pharmacy, Pocatello, ID, United States; ^6^ Department of Diagnostic Radiology and Biomedical Imaging, Magnetic Resonance Research Center, Yale School of Medicine, New Haven, CT, United States; ^7^ Biotechnology and Biomedical Center in Vestec (BIOCEV) , First Faculty of Medicine, Charles University, Vestec, Czechia; ^8^ Department of Paediatrics and Inherited Metabolic Disorders, First Faculty of Medicine, Charles University and General University Hospital, Prague, Czechia; ^9^ 3rd Medical Faculty, Charles University, Prague, Czechia

**Keywords:** extracellular vesicles, immune system, neurological and psychiatric disorders, extracellular vesicle-based therapies, regulatory agencies, pharmacokinetics, pharmacodynamics

## Abstract

The critical role of the immune system in brain function and dysfunction is well recognized, yet development of immune therapies for psychiatric diseases has been slow due to concerns about iatrogenic immune deficiencies. These concerns are emphasized by the lack of objective diagnostic tools in psychiatry. A promise to resolve this conundrum lies in the exploitation of extracellular vesicles (EVs) that are physiologically produced or can be synthetized. EVs regulate recipient cell functions and offer potential for EVs-based therapies. Intranasal EVs administration enables the targeting of specific brain regions and functions, thereby facilitating the design of precise treatments for psychiatric diseases. The development of such therapies requires navigating four dynamically interacting networks: neuronal, glial, immune, and EVs. These networks are profoundly influenced by brain fluid distribution. They are crucial for homeostasis, cellular functions, and intercellular communication. Fluid abnormalities, like edema or altered cerebrospinal fluid (CSF) dynamics, disrupt these networks, thereby negatively impacting brain health. A deeper understanding of the above-mentioned four dynamically interacting networks is vital for creating diagnostic biomarker panels to identify distinct patient subsets with similar neuro-behavioral symptoms. Testing the functional pathways of these biomarkers could lead to new therapeutic tools. Regulatory approval will depend on robust preclinical data reflecting progress in these interdisciplinary areas, which could pave the way for the design of innovative and precise treatments. Highly collaborative interdisciplinary teams will be needed to achieve these ambitious goals.

## 1 Introduction

Current management of psychiatric diseases often lacks precision in diagnosis and treatment, as such clinical management strategies do not fully address the complexity of the biological processes. Instead, diagnoses of psychiatric diseases are based on clinical presentation of neurobehavioral symptoms. In recent years, close interactions between the immune system, and the nervous system have been established in brain under physiological and pathophysiological conditions (1, 2). These interactions may offer avenues for development of new, immune-based therapies for brain pathophysiological conditions. Despite the availability of a vast range of immune-based biologics used in other inflammatory conditions (3, 4), their potential benefits for treating brain disorders have not yet been fully realized, especially in case of psychiatric diseases. The main obstacle is a legitimate concern of iatrogenically induced immune deficiency, especially in the context of brain pathophysiological conditions for which objective diagnostic biomarkers remain undetermined. A possible solution to overcome this obstacle may come from the rapidly developing field of extracellular vesicles (EVs) that could offer new diagnostic tools and more effective therapies.

## 2 Structure of this article

This review will start with a brief outline of how our understanding of the complexity of brain’s structures and functions evolved during the last twenty years. We will then provide a short overview of current pharmacotherapy for psychiatric conditions to lay a background for the remarkable potential of EVs as agents for developing fundamentally new therapies for brain disorders. We aim to elucidate how EVs can significantly enhance our understanding of neuro-immune interactions and facilitate the development of improved diagnostic biomarkers for psychiatric conditions. We will also discuss ongoing efforts to deliver new agents via specially designed EVs administered intranasally. The latter localized administration can be further improved by the expression of molecules that allow the targeting of EVs to a desired site within the brain. Taken together, these approaches may lead to more effective treatments with lower dosages and reduced risks of systemically administered therapies, e.g., immune deficiencies. As these novel therapies evolve, they will attract new requirements to be imposed by regulatory agencies to assure the safety of these treatments in the context of new findings on the distribution of fluids within the central nervous system (CNS). Addressing such considerations during preclinical development will help streamline the approval processes for these groundbreaking treatments.

## 3 Advances in understanding brain physiology and pathophysiology

About two decades ago, our knowledge of human brain structures was reflected in traditional neuroanatomy rooted in macroscopic and cellular analyses of post-mortem brain specimens and identified key brain regions and their general functions. The brain imaging *in vivo* was still of low resolution. Studies on brain functions were limited primarily to observations of changes in neuro-behavioral symptoms caused by administering pharmacological agents. Since then, remarkable advances in multiple areas of neuroscience were achieved. Most relevant to the considerations to developing new medications is the progress in brain imaging technologies, multi-omics analyses and data integration, identification of the lymphatic system in the CNS, improved understanding of fluid regulation within the brain, recognition of critical roles of glial cells and their functional interactions with neurons, diverse roles of the immune system in brain’s conditions, and the advances of our knowledge of the structure and function of EVs. These discoveries enable us to improve our understanding of the complex interactions within the brain, which contains about 86 billion neurons operating within hundreds of thousands to millions of neuronal circuitries executing diverse brain functions (5).

### 3.1 Brain imaging advances

Remarkable progress in brain imaging has enabled detailed mapping of brain structure, function, and neurochemical processes, uncovering subtle abnormalities linked to psychiatric conditions. These technologies facilitate the identification of biomarkers by correlating imaging patterns with specific symptoms, disease progression, or treatment responses, paving the way for personalized medicine in psychiatry. Progress in the field has transformed our understanding of neural structures and functions (6).

Functional magnetic resonance imaging (fMRI), developed in the 1990s, gained significance on the resolution level by employing ultra-high-field (≥7 Tesla) human MRI scanners that have pushed fMRI spatial resolution to the sub-millimeter domain. These advances make it possible to resolve functional activity and connectivity, and bring a significant promise to clinical applications in the field of psychiatry as a way to non-invasively monitor progress of a disease and effects of treatment (7–9).

Diffusion tensor imaging (DTI) maps water diffusion in white matter tracts, revealing the brain connectivity at a similarly high spatial resolution. It has greatly facilitated diverse investigations within the Human Connectome Project (10, 11).

Magnetoencephalography (MEG) provides millisecond-level temporal resolution, enabling precise tracking of neuronal activity (6). Positron Emission Tomography (PET) introduced the ability to image metabolic and neurotransmitter activity, offering a spatial resolution of 4–5 millimeters (7–9).

At a cellular level, two-photon microscopy delivers sub-micron precision for studying live neuronal structures. Super-resolution MRI pushes the limits of anatomical detail, enhancing structural imaging. Emerging techniques like time-resolved laser speckle contrast imaging allow real-time mapping of cerebral blood flow at various depths with remarkable precision. These innovations have bridged the gap between macroscopic brain imaging and microscopic neuronal activity, paving the way for new insights into brain functions and disease mechanisms.

3D imaging methodology called Clarity allows postmortem visualization of entire neuronal structure in its native context, enhancing understanding of the connectivity in an unprecedented manner (12, 13). Optogenetics and chemogenetics enable precise experimental manipulation of neuronal circuits *in vitro* in human organoids or *in vivo* in experimental animals, revealing mechanistic insights in normal or pathological physiology (14, 15). Advances in single-cell transcriptomics have also revealed new cell types and functional diversity within brain regions, thereby deepening our understanding of cellular specialization (16, 17).

### 3.2 The role of the immune system in brain functions

Not long ago, the brain was considered an immune-privileged site where immunological responses occurred only during infections. The understanding of the role of the immune system in fundamental brain functions radically changed in the last two decades, and the brain is now recognized as an immunologically highly dynamic site.

**Figure 1:**
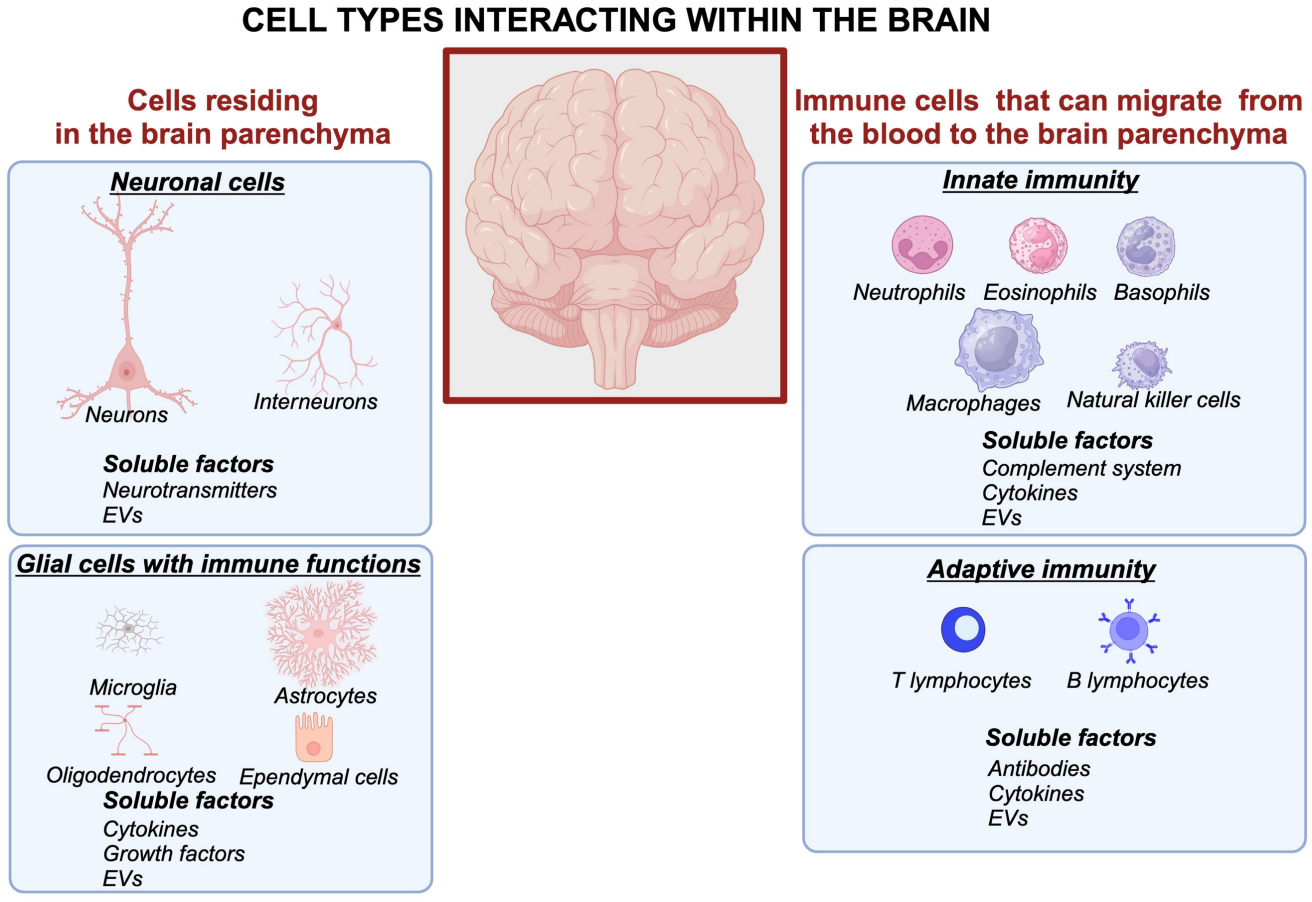
Cellular and molecular interactions within the brain form intricate networks involving neuronal, glial, and immune systems. Neurons communicate through synaptic connections and release neurotransmitters, while astrocytes and microglia modulate synaptic activity, maintain homeostasis, and respond to injury. Peripheral immune cells interact with glial cells via soluble factors such as cytokines, chemokines, and extracellular vesicles, contributing to neuroinflammation and immune surveillance. This complex crosstalk underpins brain function and its response to physiological and pathological stimuli. Created in BioRender. Kawikova, I (2024). https://BioRender.com/m42p922.

Briefly, the immune system of the brain employs four glial cell types residing within brain tissue and their functional roles are briefly summarized as follows (**Figure 1**, left panels): 1) Microglia have emerged as central players not only in responding to infections and injuries but also in synaptic pruning and neurodevelopment, shaping neural circuits during growth and even in adulthood. This population of cells is heterogenous in phenotypes that may differ depending on brain region, age, gender, and disease status (18). 2) Astrocytes, once seen as passive support cells, are now recognized as dynamic regulators influencing synaptic plasticity, fluid movement within the brain, and neuroinflammation (19–21). 3) Oligodendrocytes are responsible for myelinization of neurons (22, 23). 4) Ependymal cells form the inner lining of the ventricular system in the brain (24–26). All these glial cells contribute to neuroinflammation and produce cytokines that influence neuronal and immune responses within the brain (27) (**Figure 1**).

The body’s immune system has soluble and cellular components that arrive to the brain from the blood crossing the blood-brain barrier (BBB). A long-lasting paradigm that the BBB is impermeable has undergone a shift: it is now understood that although its permeability is tightly controlled, the BBB allows migration of the body’s immune system factors of innate immunity (neutrophils, eosinophils, basophils, macrophages, complement system, extracellular vesicles) and adaptive immunity (e.g., eight distinct subsets of B lymphocytes, antibodies, and ten distinct subsets of T lymphocytes, communicating through cytokines) and extracellular vesicles to migrate into the brain parenchyma, especially during pathological processes (28) (**Figure 1**, right panels).

**Figure 2:**
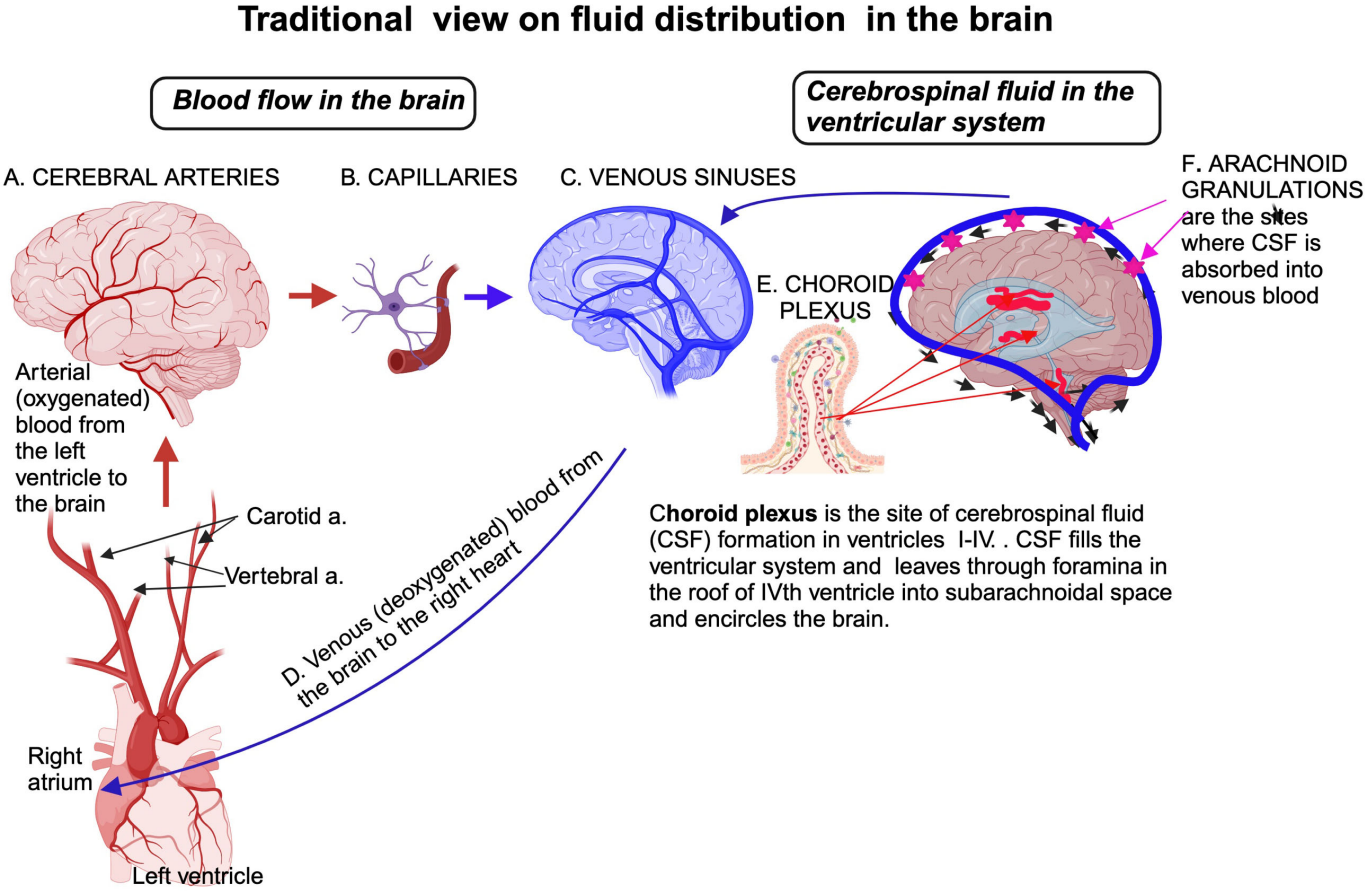
Traditional view of fluid distribution in the brain highlights two primary compartments: the vascular and CSF systems. Blood circulates through arteries, capillaries, and veins, with the BBC at the capillary level protected by astrocytic end-feet. The CSF is produced by the choroid plexus within the ventricles, circulates through the ventricular system, and flows into the subarachnoid space surrounding the brain and spinal cord. CSF is continuously reabsorbed into the venous circulation via arachnoid granulations in the sagittal venous sinus, maintaining homeostasis and clearing waste products. Created in BioRender. Kawikova, I (2024). https://BioRender.com/q62s356.

**Figure 3:**
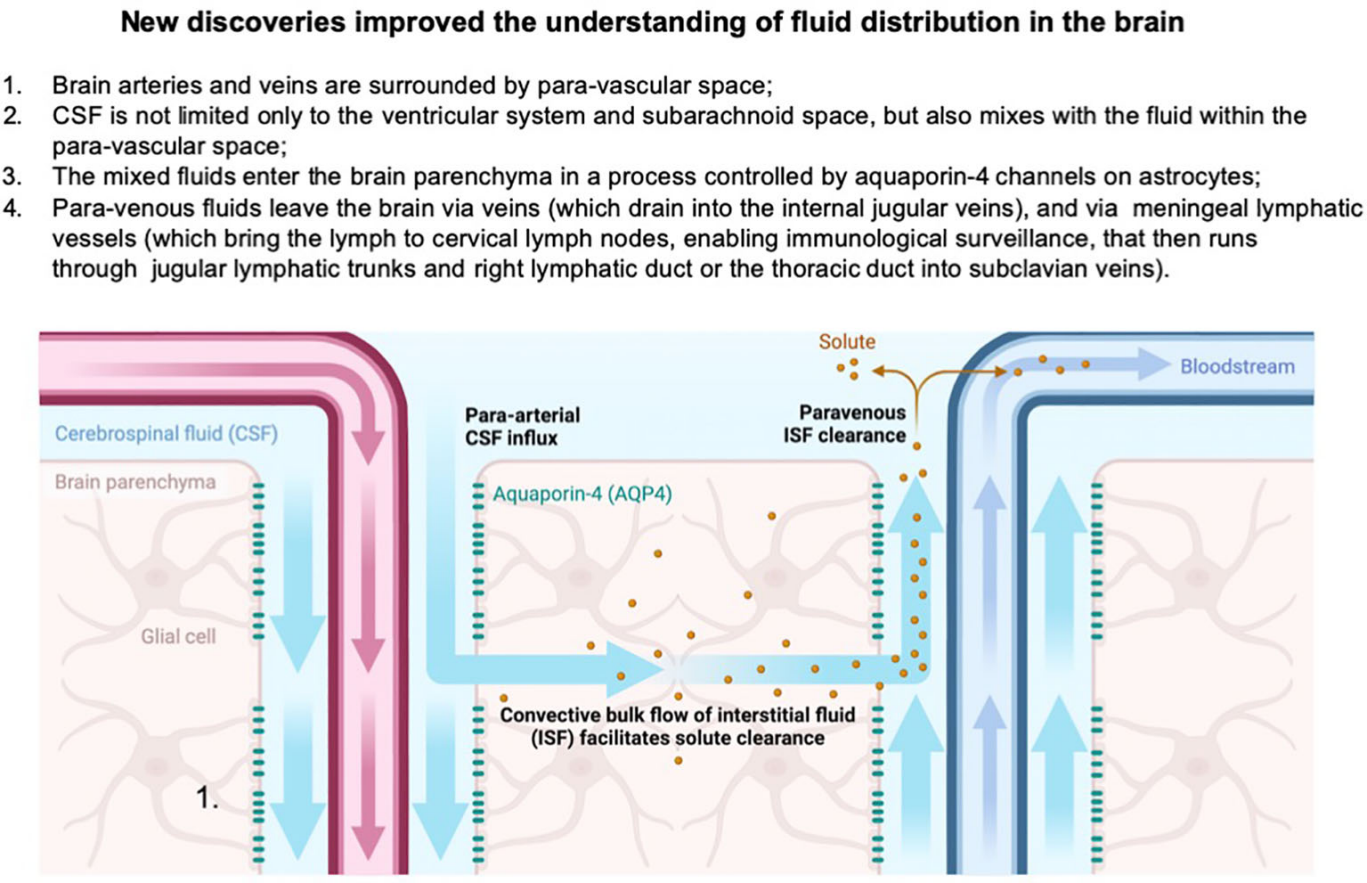
Paradigm shifts in the understanding of brain fluid dynamics highlight the discovery of the paravascular space, where CSF exchanges with interstitial fluid in the parenchyma, forming a glymphatic clearance pathway. This mixing facilitates the removal of metabolic waste from the brain. Additionally, the identification of meningeal lymphatic vessels reveals an alternative drainage route, where a portion of the brain’s fluids exits into cervical lymph nodes and subsequently drains into the subclavian veins, providing a critical link between central nervous system fluid dynamics and peripheral immune function. Created in BioRender. Kawikova, I (2024). https://BioRender.com/u61z109.

The traditional view on the role of the immune system and fluid distribution (**Figure 2**) was fundamentally altered after the discovery of *meningeal lymphatic vessels and glymphatic* system enable a portion of fluids within the brain to reach cervical lymph nodes for immune surveillance; in this way, the brain is connected to the peripheral immune system that plays roles in both normal and pathological physiology (29) (**Figure 3**).

The gut-brain axis has been recognized as a crucial communication pathway between the gut microbiota, mucosa-associated lymphoid tissue, and the brain, influencing mood, stress responses, and cognitive function 34. More recently, the lung-brain axis (30) and liver-brain axis (31) have been identified, though these axes remain less well characterized.

**Figure 4:**
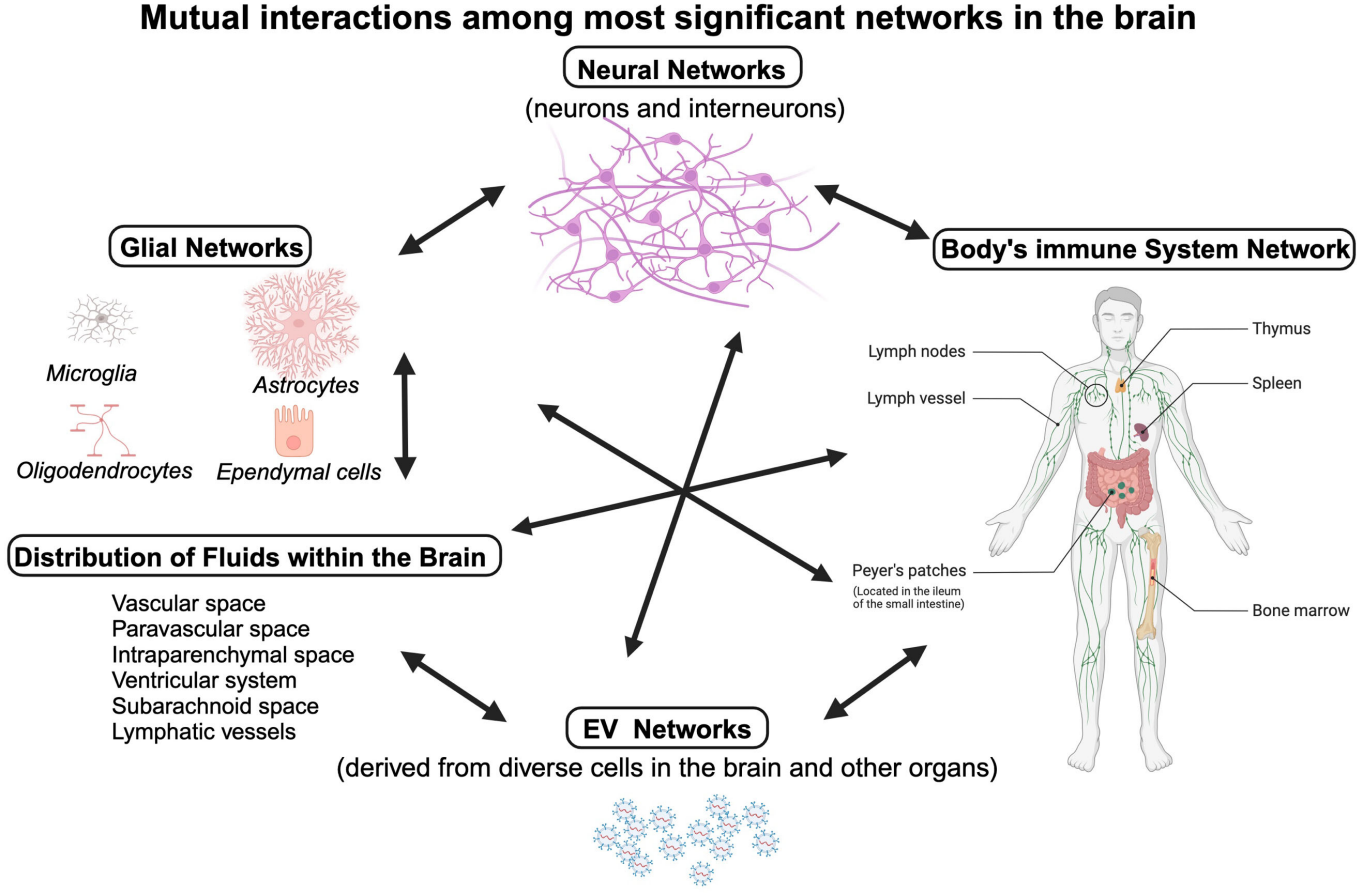
Homeostasis in the brain parenchyma is critical for fundamental functions, e.g. action potential propagation and myelination. It involves close interactions between four networks: the neuronal network (neurons, interneurons, and neurotransmitters at neuronal synapses), the glial network (consisting of astrocytes, microglia, oligodendrocytes and ependymal cells), the body’s immune cells that may infiltrate the brain parenchyma (cytokines and antibodies, innate and adaptive immunity cells), and the EVs networks. These networks communicate with each other, affect interchangeably and can be also affected by interstitial fluid tightly controlled by a web of astrocytes. Individual cells of these networks can alter biochemical composition of the fluids, as they release cytokines and metabolic products into their surroundings. In addition, the movement of fluids within the brain depends on circadian cycle (83). Created in BioRender. Kawikova, I (2025). https://BioRender.com/m42p922.

The body’s immune system and the brain’s immune system interact bidirectionally (**Figures 1**, **4**). For example, in patients with an autoimmune condition, such as lupus, autoantibodies were shown to alter the activity of neurons in the hippocampus and result in neurobehavioral symptoms (32, 33). Other reports indicate altered numbers of regulatory T lymphocytes in psychiatric patients (34–36), suggesting a vulnerability to autoimmune diseases. Many studies reported increased expression of inflammatory markers in the brains of patients with psychiatric diseases or elevated levels of cytokines in the blood of such patients (34, 37, 38).

### 3.3 Fluids in the brain

Water content in the brain represents about 75-80% of its weight, placing the brain among the organs with the highest water content. Historically, medical textbooks described four fluid compartments within and around the brain: the intravascular space, the intercellular fluid in the parenchyma, the cerebrospinal fluid (CSF) in the ventricular system, and the arachnoid space. It has been known for a long time that the border between capillaries and brain parenchyma is guarded by the BBB of endothelial cells with tight junctions and astrocytes, viewed as mostly impermeable (39). Water movement *between vascular* sp*ace and brain tissu*e was believed to occur primarily through passive diffusion. The CSF’s formation in the choroid plexi of all intra-cerebral ventricles was recognized. It was believed that the flow of the CSF is unidirectional, moving from its site of formation, filling the intracerebral ventricular system, and leaving via openings in the roof of the fourth ventricle (foramina Lushka and Magendie) into the subarachnoid space surrounding the brain and spinal cord. From here, the CSF was assumed to travel solely to the vascular system through the primary site of absorption, arachnoid villi, and granulations in the superior sagittal sinus through a pressure-dependent process controlled by unidirectional valves (**Figure 2**).

Fundamental discoveries in recent years have changed these paradigms. First, the discovery of aquaporin channels in end-feet of astrocytes within the BBB revealed that water movement across the barrier is not a passive diffusion but an active, regulated process where aquaporin-4 (AQP-4) channels respond to extracellular osmolarity within the brain parenchyma (40, 41). Second, CSF was noted to move from the ventricular system and subarachnoid space into brain parenchyma and leave through the paravascular system where the movement of fluid is facilitated by arterial pulsing. In para-arterial space nutrients are delivered and in para-venous space, waste products are removed from the interstitial fluid surrounding neuronal and glial cells within brain parenchyma (42). This process is again dependent on AQP-4 channels on astrocytes (42). This fluid compartment was named the glymphatic system as it resembles lymphatic drainage in other places of the body and is distinguished by the active role of the glial subsets, astrocytes. Important roles of this system in neurodegenerative conditions became quickly recognized (43). Third, the discovery of lymphatic vessels in the meninges revealed a new absorption pathway for the CSF leaving the brain and connected the brain lymphatic system with the whole body’s systemic system (44). Fourth, a critical role of differential fluid movements and waste removal during the circadian cycle was shown, thereby pointing to the critical role of sleep disturbances in neurodegenerative conditions (45–47) *(* **Figure 3**).

### 3.4 Extracellular vesicles

*EVs* are particles released from cells. A lipid bilayer envelops them and they cannot replicate independently (48). EVs carry bio-active molecules and are critical for inter-cellular communication (48). Until recently, they were classified according to their size and biogenesis into two main groups: exosomes (150 nm EVs) originating in the endosomal pathway and released outside of cells when multi-vesicular bodies fuse with cell membrane, and micro-vesicles (100-1,000 nm) that are generated by outward budding of the cell membrane. With growing knowledge, this classification became confusing and therefore discouraged. Individual studies on EVs should provide details about the EVs population they investigate, including their biogenesis, sizes, density, and cargo (48).

A significant milestone in the EVs field occurred in 2007, when Jan Lotvall’s group demonstrated the presence of the functional ribonucleic acid (RNA), including messenger RNA and microRNA in cell-derived EVs, at that time called exosomes (49). This finding was groundbreaking and suggested that EVs are a protective vehicle for the transport of nucleic acids, proteins, and lipids and mediate intercellular communications among cells in the vicinity or at a distance. With the discovery of small inhibitory RNAs, it became apparent that a potent, unknown regulatory system exists in our body. This new concept opened many avenues to understand fundamental biological regulations (50). Since then, tremendous progress has been made in methodologies that enable EVs isolation from biological fluids and in insights into the differences in EVs in various disease conditions.

In neurons, EVs were described for the first time already in 1955 (51). EVs are released by all cells within the brain and significantly impact the brain’s pathophysiology (50). A recent update on EV’s role in CNS physiology and neurological diseases has been reviewed (52).

EVs derived from glial cells (**Figure 1**, left) showed their roles in intercellular communications and mutual critical impacts on essential functions and their roles are briefly summarized below (52–55). Microglial cells (56) release EVs that regulate *synaptic pruning and differentiation*, a critical function for proper brain development and functioning. Microglial EVs carry complement proteins (C1q, C3, C4), which tag synapses for elimination, ensuring synaptic refinement. This function is dysregulated in neurodevelopmental disorders (57, 58). Microglia also engage in neuroinflammation. For example, when the microglial cell line is stimulated with lipopolysaccharide, their released EVs contain a higher concentration of interleukin (IL)-6 and tumor necrosis factor (TNF) alpha (59). When microglia are incubated with EVs derived from the serum of patients with autism spectrum disorder compared to normotypic control subjects, they release significantly higher amounts of IL-1 beta (60). *In vivo*, microglia-derived EVs reduce post-stroke myelin damage via protective effects on oligodendrocytes (61). Another glial lineage, astrocytes, which provide metabolic and neuroprotective support to neurons, carry proteins and microRNAs that protect neurons from oxidative stress and promote their survival, as indicated by the protective effects of EVs derived from astrocytes exposed to hypoxia (62), or regenerative effects on neurons exposed to traumatic brain injury (63). Astrocyte-derived EVs can also attenuate or impair (64) the permeability of the BBB (65, 66). Oligodendrocytes, the myelin-producing cells, alter EV’s content when exposed to endoplasmic reticulum stress (67, 68), and can provide protective effects on neurons (69), partly via secretion of ferritin heavy chain (70). Not surprisingly, oligodendrocyte-derived EVs play important roles in the pathogenesis of multiple sclerosis (71). Studies on EVs derived from ependymal cells only began to emerge. Nonetheless, the first study indicates their important role in cilia functions (72) that affect movements of fluid in the ventricular system, which is believed to play critical roles in neurodegenerative processes.

EVs with diverse cargos are formed during activities of the body’s immune system (**Figure 1**, right) (73). Since EVs cross BBB bidirectionally (74), EVs from the body’s immune system can modulate activities within the brain, and vice versa. In summary, EVs derived from the brain’s or the body’s immune cells can propagate or reduce inflammatory signals depending on their cargo and target cells.

The relevance of EVs to psychiatric conditions was established by experiments where EVs isolated from the peripheral blood of schizophrenic patients (75) were transferred into mice, which then exhibited decreased pre-pulse inhibition, acoustic startle response, tail suspension, and elevated plus maze and increased score on open field test (75). Mice receiving EVs from patients with major depressive disorder (MDD) (76) experienced depressive behavior such as increase forced swimming, tail suspension and the novelty suppressed feeding. Elucidating EVs cargos in patients with different types and subtypes of psychiatric conditions will likely lead to the design of essential diagnostic tools, as suggested by many ongoing studies (77, 78). A deeper understanding of the functional impacts of EVs and their cargo and the formulation of protocols to design EVs in a laboratory setting have stimulated considerable enthusiasm for developing new therapeutic tools for human brain diseases.

We are now at the frontier of a new era in which EVs-based pharmacotherapies bring promises of a fundamental shift in how drugs are delivered and how diseases are treated. These nano-scale vesicles, naturally secreted by cells, offer unparalleled potential for precision medicine by enabling targeted delivery of therapeutic molecules, crossing biological barriers like the BBB, and reducing systemic side effects. To provide a foundation for understanding this fundamental shift, let us look at historical milestones of medications used in psychiatry and appreciate the need for the improvement that EVs-based therapies can offer.

## 4 Historical milestones in pharmacological interventions for psychiatric conditions that led to existing therapeutics

The management of psychiatric conditions started to involve pharmacotherapeutic approaches in the second half of the 19th century (79), replacing physical restraint and similar methods after the introduction of morphine, potassium bromide, chloral hydrate, hyoscine, or paraldehyde. Several milestones occurred in developing medications used to treat neuropsychiatric conditions. During the first half of the 20th century, the introduction of penicillin, nicotinic acid (niacin, vitamin B3), or thiamine (vitamin B1) helped to effectively modify outcomes of dementia due to syphilis, pellagra, or alcohol abuse disorder (79). Also, in the early 1900s, sedative medications - barbiturates - were commonly used; however, these sedative medications are currently limited to the management of epilepsy and anesthesia. During the second half of the 20th century, neuropharmacology truly flourished. In the 1950’s, the discovery of chlorpromazine led to the development of antipsychotic and anti-depressive medications. In the 1960’s, benzodiazepines were introduced for anxiolytic disorders; lithium was re-introduced for the treatment of mania in bipolar disorder, and high-potency antipsychotic medications started to be used for acute psychosis and schizophrenia. The 1980’s witnessed the development of selective serotonin reuptake inhibitors (SSRIs), with fluoxetine becoming one of the most well-known medications for depression. The late 1980’s and 1990’s also witnessed the rise of second-generation (atypical) antipsychotics, such as clozapine and risperidone, which aimed to treat schizophrenia with fewer side effects in comparison with treatment with older antipsychotics.

The discoveries of pharmacotherapeutics led to the theory that psychiatric disorders are primarily caused by the dysregulation of a single neurotransmitter, known as the “monoamine hypothesis” or, more broadly, “neurotransmitter imbalance theory.” This theory has been particularly prominent in explaining mood disorders, especially depression, where it posits that the disorder results from imbalances in neurotransmitters like serotonin, norepinephrine, and dopamine. This picture, however, is now viewed as oversimplified and not substantiated, especially in the light of recent progress in neuroscience (80).

## 5 Need for further therapeutic advancement

Despite these significant advances in the pharmacotherapy of neuropsychiatric conditions, important challenges remain, such as lack of efficacy in subpopulations of patients that was estimated to occur in at least thirty percent of psychiatric patients (81). Also, notable side effects may reduce compliance of patients (81). Substantial side effects include the metabolic syndrome with a weight gain, cognitive impairment, sedation, sexual dysfunctions, and others. Patients on high-affinity anti-psychotics may suffer extra-pyramidal syndromes, such as acute dystonia, akathisia, parkinsonism, or tardive dyskinesia. Individuals treated for Parkinson’s disease may be affected by L-DOPA-induced dyskinesia. In addition, medications for major depressive syndrome have a prolonged onset of effects lasting several weeks after starting the medication (81).

Existing evidence links immune system dysregulation to the pathophysiology of disorders such as schizophrenia, depression, and autism spectrum disorders. Immune-based therapies, including cytokine modulation, anti-inflammatory agents, and immune checkpoint inhibitors, hold promise for targeting the underlying neuroinflammation and restoring immune balance, potentially offering novel avenues for treatment where traditional psychiatric medications may fall short (81).

## 6 Immune-based therapies for psychiatric conditions: obstacles and promises

### 6.1 Obstacles

#### 6.1.1 Iatrogenically-induced immune deficiency

A significant obstacle for clinicians in employing immune modulators is the concern that immune inhibition renders patients vulnerable to infectious microorganisms that may worsen brain function. Localized and targeted therapeutics with minimized systematic effects could resolve this legitimate concern.

An example of a successful bypass of immune function inhibition is the switch from systemic to localized treatment in managing bronchial asthma. Asthma attacks and exacerbations used to be treated with oral or intravenous corticosteroids. With their more prolonged use, systemic effects of Cushing syndrome appear. The introduction of inhaled steroids reduced the side effects remarkably as several forms of inhaled steroids and new devices for delivery, such as metered dose inhalers (81). Today’s guidelines include long-term treatment with corticosteroids that limit airway inflammation and prevent airway remodeling and may be formulated as dual therapies with beta-agonists that relax airway smooth muscle (81).

The historical example may serve as a motivation for developing localized and targeted treatments for psychiatric conditions: intranasal delivery of multiple compounds has been shown to have beneficial effects at the pre-clinical and clinical levels, including growth factors, vitamins and metabolites, cytokines, and immunosuppressants (81). Thus, localized administration and targeting within the brain may eliminate the concern about significant iatrogenic immune deficiency (81).

#### 6.1.2 Lack of objective diagnostic tools in psychiatric conditions

Another major obstacle to developing immune-based therapies for psychiatric conditions is the imprecision of the current diagnostic process in psychiatry, which is mainly based on neuro-behavioral symptoms because objective biomarkers are not available for clinical application. To address this issue, it is crucial to prioritize the development of objective diagnostic tools that reflect the pathophysiology of diseases rather than behavioral phenotypes. As outlined in **Figure 4**, interactions among neuronal, glial, and immune networks on the backdrop of changes in fluid distribution in brain parenchyma are very complex.

EVs-based diagnostics hold significant promises for the early detection and monitoring of diseases, but they are currently in the developmental and validation stages. Significant progress was made in this area when Mercy BioAnalytics received Breakthrough Device Designation from the U.S. Food and Drug Administration (FDA) for their Mercy Halo™ Ovarian Cancer Screening Test in May 2024. This test utilizes EVs-based liquid biopsy technology for the early detection of ovarian cancer in asymptomatic, postmenopausal women (81). Another achievement is the use of EV protein-based blood tests for the early detection of multiple cancers, including pancreatic ductal adenocarcinoma, ovarian, and bladder cancers (81).

A breakthrough in the diagnosis of psychiatric conditions is likely to come from multifaceted, longitudinal studies of sizeable groups of patients suffering from distinct psychiatric diseases. Extracellular vesicles (EVs) carry cell surface markers of cells of their origin, and thus, it is feasible to isolate brain-derived EVs from readily available blood samples. Detailed analyses of the content of brain-derived EVs by -omics techniques (e.g., transcriptome, microRNA, proteomic, or lipidomic profiles) promise to reveal pathophysiological patterns and improve diagnostic approaches in psychiatry. For example, the EVs microRNA profile can distinguish between psychiatric conditions, such as major depressive disorder, Attention-Deficit-Hyperreactivity Disorder, and Anxiety disorder (82).

### 6.2 Advances related to EVs-based therapies for CNS conditions

Currently, there are three types of EVs-based therapies developed: naturally-produced EVs, artificial EVs and hybrid EVs. Advantages and shortcomings of each of these types are summarized in **Table 1** below.

**Table 1:** Summary of advantages and disadvantages of different types of EVs.

Type of EVs	Advantages	Disadvantages	References
*Natural*	Biocompatibility, immune system compatibility, natural targeting properties	Limited scalability, heterogeneity, potential safety concerns	(142–145)
*Artificial*	Customizable properties, scalable production, controlled composition	Potential immunogenicity, lack of natural targeting, challenges in mimicking biological functions	(146–148)
*Hybrid*	Combines the advantages of natural targeting with artificial customization, increased stability and efficiency	Complex manufacturing processes, potential for unforeseen interactions between components	(149, 150)

The realization that EVs are primarily responsible for the regenerative power of stem cells opened new therapeutic avenues because *naturally produced EVs* reduced concerns about immunogenicity and the host’s rejection of cell-based therapies (84). Thus far, the EVs has not been demonstrated to exert immunogenic properties. The sources of natural exosomes for experimental treatments have included mesenchymal stromal cells (MSC), Wharton Jelly stem cells (SC) (85–87), umbilical cord SC (88–93), neuronal SC (89, 94), and adipose tissue-derived SC (95–99).

The stem cell-derived EVs contain a variety of molecules (including neurotrophic factors and anti-inflammatory cytokines, such as tumor growth factor beta or interleukin-10) that may mediate their neuroprotective effects (100). Interestingly, EVs derived from different sources exhibited beneficial effects in multiple experimental models of brain diseases despite the lack of standardization among the experimental protocols. The beneficial effects were shown in ischemic brain injury (101–104), traumatic brain injury (105–108), Alzheimer’s disease (109–112) (113) (114), Parkinson’s disease (94, 115–118), amyotrophic lateral sclerosis (119), multiple sclerosis (120), spinal cord injury (84, 121–124), major depressive disorder (125, 126), schizophrenia (127, 128), substance abuse (125, 126, 129, 130), and anxiety (131).

The need for standardized EVs production with well-defined cargo led to the development of a second type of innovative therapeutic EVs: *artificial EVs* (132). Initially, artificial exosomes were designed as a vehicle for curcumin, a substance with anti-inflammatory effects. Intranasal administration of curcumin-laden exosomes resulted in protective effects in a model of experimental autoimmune encephalitis (133), Alzheimer’s disease (134), Parkinson’s disease (135), and brain tissue ischemia (115). In their Parkinson’s disease model, Haney et al. experimented with multiple ways of loading an enzyme catalase into EVs, and their intranasal administration resulted in significant neuroprotective effects (136, 137). EVs represent a delivery vessel for microRNA that then impacts specific protein transcription and translation at the site of an injury. An example of such an approach includes experiments where EVs-bearing microRNA 181a directed to the ischemic site via surface binding protein reacting with receptors for advanced glycation end-products expressed highly on cells in the ischemic brain. Intranasal delivery of the EVs reduced the infarct size and showed how localized EVs administration can target the injury site precisely (138).

By combining the advantages of the above-mentioned two EVs types, a third type was created, the *hybrid EVs.* Hybrid EVs help overcome some inherent problems, e.g., complex technologies for EVs isolation, their low yield, heterogenous content, and difficulties targeting them to desired sites. For example, the facilitation of EVs transcytosis across the BBB can be achieved by expressing transferrin on isolated EVs that then reacts with transferrin receptors on endothelial cells (139). The current activities related to hybrid EVs are summarized well in recently published reviews (140, 141).

## 7 Regulatory agencies and EV-based therapies for CNS disorders

No EVs-based therapy has been approved by the United States Food and Drug Administration (FDA) or the European Medicines Agency (EMA) yet. The FDA’s and the EMA’s approval process for any therapeutic agent demands robust evidence encompassing its safety, efficacy, and quality, necessitating meticulous preclinical and clinical data adherence to regulatory standards. Significant progress is being made in this field, with EVs-based therapies undergoing clinical trials to assess their safety and efficacy (see below).

### 7.1 General requirements

Full approval for a new therapeutic agent requires several well-defined steps involving in initiating clinical trials and ultimately resulting in full regulatory approval. First, sound preclinical research should provide safety, efficacy, and proof-of-concept data. The process for authorization to begin a clinical trial requires an Investigational New Drug (IND) Application (FDA)/Clinical Trial Application (CTA) (EMA). To obtain the authorization, documents that demonstrate safety and efficacy from animal studies need to be provided, as well as manufacturing information about the source of EVs (e.g., MSCs, immune cells), isolation and purification protocols, characterization of EVs (e.g., size, cargo, purity, potency) and compliance with Good Manufacturing Practices (GMP). Further documentation includes a clinical protocol with a detailed plan for Phase 1 trials (e.g., objectives, patient population, dosage, and endpoints) and risk assessment (e.g., immunogenicity, potential contamination, or tumorigenicity). During Phase 1 trials, safety, tolerability, and pharmacokinetics are assessed in a small group of healthy volunteers or patients. In Phase 2 trials, efficacy and safety in a larger patient population is established. In Phase 3, efficacy is confirmed, side effects are monitored, and the new therapy is compared to standard treatments in a large, diverse patient group. To obtain full approval, a Biologics License Application (BLA) (FDA)/Marketing Authorization Application (MAA) (EMA) needs to be submitted. The application requirements integrate results from all clinical trial phases showing safety, efficacy, and benefit-risk profiles. In regard to manufacturing data, GMP compliance for large-scale EVs production and evidence of batch-to-batch consistency need to be documented and provided. Furthermore, detailed labeling, including dosage, indications, contraindications, and post-marketing plans (e.g., proposals for Phase 4 studies to monitor long-term safety and effectiveness) are required. Finally, in Phase 4, long-term safety, rare side effects, and effectiveness in the general population are monitored.

Concerning EVs-based therapeutics’ development, the variability of EVs source may lead to inconsistent treatment effects; and EVs will need to be characterized in detail (e.g., size, cargo, potency) to ensure quality. Additionally, translating EVs production to GMP-compliant, large-scale processes can be challenging. Addressing concerns about potential effects on fluid distribution within the brain or effects on immune cells, especially those in cervical lymph nodes, will need to be considered. Since EVs-based therapies are a novel class, guidelines for their approval are still developing. However, we can anticipate that regulatory agencies will hold investigators to the highest possible standards and will require considerations arising from novel scientific discoveries.

While EVs-based therapeutics are promising for future medical applications, they are currently only investigational. Ongoing research and clinical trials will provide more insights into their potential, and regulatory approvals will depend on the outcomes of these studies. The steps for approving EVs-based therapies are similar in structure to those for other biologic therapies like cell-based therapies (e.g., MSC-based or CAR-T therapies) and gene therapies, as they all involve rigorous preclinical and clinical evaluations to ensure safety, efficacy, and quality. However, key differences exist due to the unique characteristics of EVs, particularly concerning their heterogeneity, cargo composition, and safety. More standardized protocols and guidelines will help bridge these gaps as the field matures.

We will discuss the key considerations in this process and review clinical studies designed to establish feasibility and safety, some of which also involved compassionate use during the COVID-19 pandemic.

### 7.2 Minimizing off-target effects

This goal can be achieved by restricting EVs effects to the brain by choosing the most relevant route of EV administration. Further reduction of EVs concentration may be achieved by targeting EVs to a specific site within the brain.

#### 7.2.1 Route of administration

EVs-based therapies are being explored through various administration routes, each with distinct advantages and disadvantages. One of the most attractive approaches is *intranasal administration* (137), which represents a non-invasive approach with direct delivery to the brain. Intranasal administration is associated with rapid onset of effects and limited systemic exposure. The disadvantages of this approach are a limited dosage volume, variable absorption, and mucociliary clearance that may reduce EVa availability. *Oral administration* is also non-invasive and easy to apply. While considerations about the degradation of EVs in the stomach may not be a problem^160^, the first-pass metabolism in the liver significantly reduces the bioavailability of the absorbed EVs. In addition, the absorption in the gastrointestinal tract may vary among individuals or with a change in diet. The widespread systemic distribution and well-controlled dosage are achieved by *intravenous* a*dministration* of EVs although this procedure is invasive, and the consequent wide distribution of EVs may be associated with an Increased risk of their off-target effects. The less common route, *direct administration to the brain*, could deliver EVs to the desired brain region. However, this direct procedure is invasive and requires a trained neurosurgeon, a therapy approach that deters patient willingness to accept. In summary, intranasal administration of EVs-based therapies offers a promising balance between efficacy and safety for CNS targeting. It can provide direct brain delivery while remaining non-invasive, though challenges like limited dosage volume and mucociliary clearance exist (75, 137, 151).

#### 7.2.2 Enrichment of EVs in targeted areas

One significantly efficient approach to EVs targeting specific brain regions can be achieved through the surface modification of the EVs by attaching ligands or peptides that bind specifically to receptor expression on BBBs or in particular brain regions. In an experimental setting, engineered EVs expressing rabies virus glycoprotein (RVG) given intravenously delivered small inhibitory RNA to cells expressing cholinergic receptors in the brain (a target of RVG) and reduced the expression and protein production of the targeted gene (152). In another study, the attachment of transferrin and low-density lipoprotein receptor ligands to EVs enhanced their penetration through the BBB and showed promising results in Alzheimer’s animal models (153). Cooper et al. demonstrated the use of EVs loaded with alpha-synuclein siRNA to target dopaminergic neurons in Parkinson’s disease models resulted in reduced symptoms in the EV-treated models (154). The development of targeted EVs for brain therapy is advancing rapidly, with several promising strategies that include surface modification with ligands or other targeting engineered for cell specificity. These efforts can potentially lead to the design of EVs-based therapies that could improve outcomes of neurodegenerative or neuroinflammatory diseases with reduced side effects.

### 7.3 EVs pharmacokinetics

**Figure 5:**
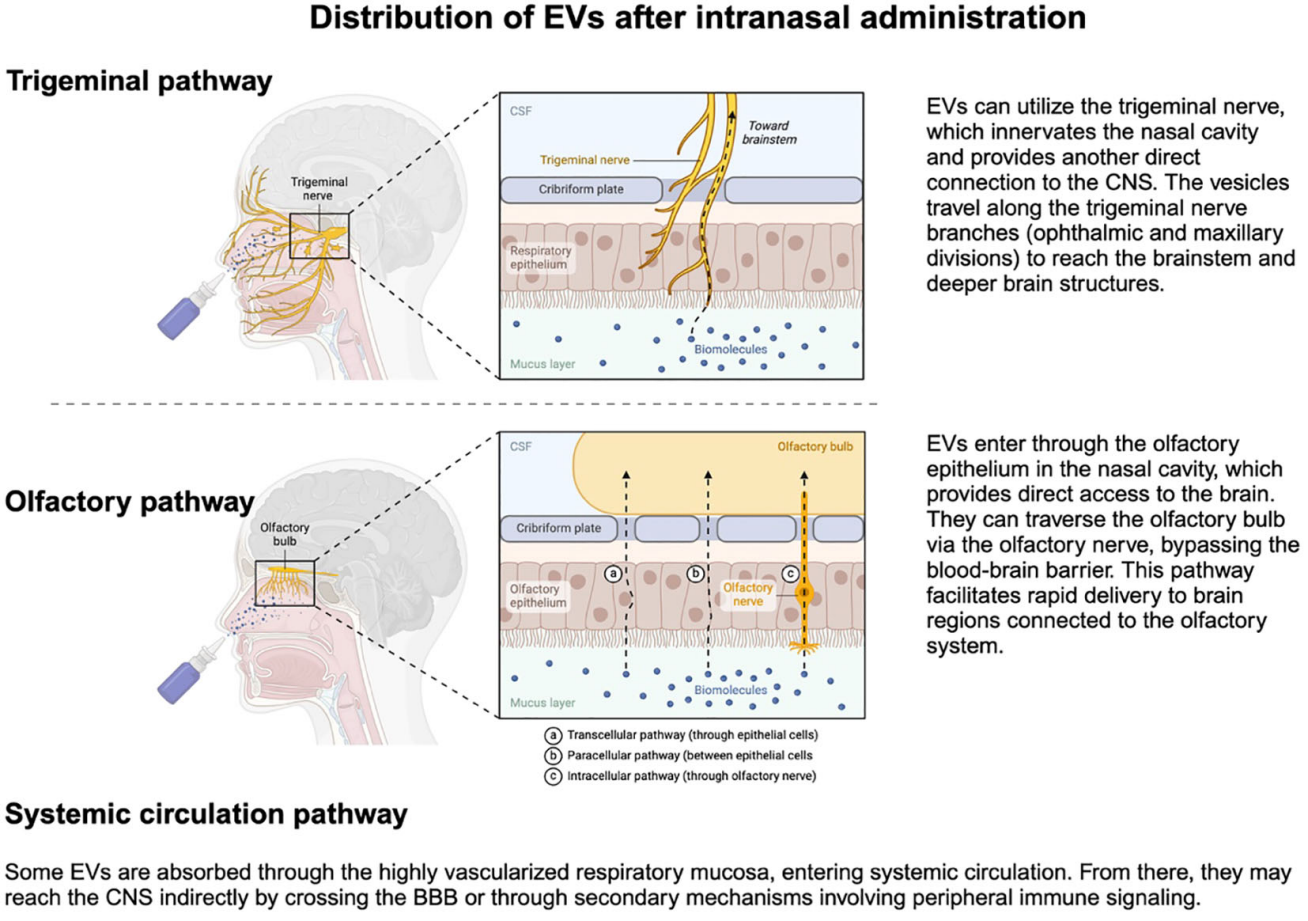
Intranasal administration of EVs enables their transport through the trigeminal and olfactory pathways, and partially also through the nasal mucosa into systemic circulation, reaching the BBB, and delivering therapeutic cargo to the central nervous system. Created in BioRender. Kawikova, I (2024). https://BioRender.com/s33f934.

Intranasal administration of EVs-based therapy (**Figure 5**) offers several advantages that include: 1) bypassing the first-pass effect in the liver that significantly reduces bio-availability of orally administered medications; 2) bypassing the BBB, at least in part; 3) low primary volume of distribution that reduces the amount of needed EVs to achieve therapeutic concentrations; and 4) non-invasiveness of intranasal administration (137).

Pharmacokinetic considerations of intranasally administered EVs for brain diseases begin with the formulation of these vesicles (including the surface molecules that affect the preferential enrichment in target areas) and their cargo. The size and molecular weight of such vesicles will determine their physicochemical properties and, in turn, their distribution (155).

Upon intranasal administration (**Figure 5**), most EVs arrive in the brain (156, 157). In the intranasal cavity, EVs comes in contact with epithelial cells, which they cross by transcytosis (100). This process is opposed by the clearance of particles by cilia on epithelial cells and by the mucus on the surface of the nasal cavity that contains degrading enzymes, such as P450, peptidases, and proteases (100). How these factors can affect EVs bioavailability will be critical to determining the effective therapeutic concentrations of each EVs type and its cargo. Low bioavailability was previously reported for drugs delivered by liposomes (158), which was overcome by polyethylene-glycol coating and was recently employed also for designing intranasal EV (159). Also, the transcytosis process through epithelial cells is likely to be affected by the EV’s physicochemical properties: blood flow through nasal mucosa, for example, is reduced in a cold environment or increased during inflammatory conditions, such as rhinitis. From the nasal cavity to the brain parenchyma, EVs travel in the perivascular space (156) and along the sensory nerves, mainly cranial nerve I (olfactory), and V (trigeminal) (156).

A critical pharmacokinetic factor is the volume of distribution required for the calculation of the loading dose of any therapeutic agent. Fundamental discoveries in recent years (**Figures 3**, **4**) changed paradigms about the communication between different fluid compartments in the brain, the CSF formation (160), unique aspects of lymphatic drainage, and the role of astrocytes in propelling fluid movements. The water content of the brain is about 75-80%, which places the brain among the organs with the highest water content. Fluid compartments within the brain consist of the intravascular space, intercellular fluid in the parenchyma, CSF in the ventricular system and subarachnoid space, and lymphatic vessels. Communications between the individual compartments are tightly regulated to ensure the delivery of oxygen and nutrients, removal of waste products, and the maintenance of immune surveillance while preventing the build-up of pathologically high pressures in the confined space within the confines of the bony skull (161). These regulatory controls will likely be affected by the pathogenesis of a brain disease to be treated, as well as the bioactive cargo of selected EV-based therapeutics. Given the variety of EV sizes and molecular weight of their cargo, it will be essential to understand how EVs move within the fluid compartments.

Another essential pharmacokinetic factor is the determination of the half-life and clearance of each therapeutic agent, which, in turn, allows the assessment of how the therapeutic dose of the agent can be maintained. Clearance of intranasally administered EVs involves two routes: 1) absorption of EVs in the nasal mucosa, which brings EVs to the systemic circulation, and from there, they are cleared by kidneys and liver; and 2) EVs enter the CNS via olfactory and trigeminal pathway (**Figure 5**) and are dispersed in the CSF, which can then mix with the fluid in paravascular space. The EVs-containing CSF is then absorbed by arachnoid granulations into venous blood of the sagittal sinus (**Figure 2**) or meningeal lymphatic vessels (162) that transport the EVs first to cervical lymph nodes and then to subclavian veins (163). In addition, EVs that ended up in brain parenchyma can be phagocytosed by microglial cells and perivascular macrophages or undergo transcytosis in endothelial cells. EVs may also be degraded enzymatically in the nasal mucosa or the brain parenchyma.

Although arguably important, the impact of EVs-based therapeutics on ependymal cilia (164, 165) and on the activity of the glymphatic system (which may also be independently affected in certain neurobehavioral diseases) (166, 167) has yet to be determined.

### 7.4 EVs pharmacodynamics

An in-depth understanding of the pharmacodynamics of EVs-based therapeutics is crucial for their practical applications in treating brain diseases. EVs offer a promising avenue for drug delivery due to their ability to transport bioactive cargo across biological barriers, including the blood-brain barrier. However, to maximize their therapeutic potential, it is essential to comprehensively evaluate their pharmacodynamic properties, including dose-response relationships and variability in bioactive cargo.

Elucidation of their dose-response relationships is fundamental for optimizing the efficacy of EVs-based intranasal therapies. This requirement involves determining the appropriate concentration of selected vesicles and the components of their bioactive cargo. Precise dosing of both components in intranasal therapeutics is critical to ensure effective therapy of brain-located pathologies.

Currently, MSC-derived EVs are sourced from multiple tissue types, contributing to significant variability in dosing. A recent report highlighted this variability, stating that intranasally administered MSC-derived EVs doses range from 0.02 to 600 × 10^10^ particles (168).

Standardization of EV sources, their dosing, and detailed characterization of EVs cargo components (which may differ depending on the physiological or pathological condition of the parent cells) will be essential for optimizing the pharmacodynamic effects of EVs-based therapeutics.

### 7.5 Manufacturing

With regards to manufacturing therapeutic EVSs, many challenges need to be faced, including the consistency of an EVSs source and standardization of their size (nanovesicles or also micro-vesicles), content (depending on their source, they may contain variable mixes of proteins, mRNA, microRNA, lipids), and dosage (typically expressed as particles per ml or content of protein per vesicle or as an activity in a biological assay). The doses of EVSs are then adjusted in clinical trials to “concentration of exosomes”/kg of patient weight and their therapeutic safety is established at Phase I clinical trials, using Fibonacci sequence to establish Maximal Tolerated Dose (MTD), which can be further adjusted based on clinical study parameters, e.g. PK/PD. For large-scale production of EVSs, additional important factors need to be resolved to assure their scalability, stability and storage of EVSs (169–171).

### 7.6 Safety

Safety considerations are paramount in successfully passing the FDA and EMA approval process for new therapeutics. Consequently, detailed preclinical experiments and clinical trials in human subjects are required to assess the safety, efficacy, and quality of a new therapeutics. One crucial step in developing EVSs-based therapies is achieving cell-specific targeting, which enhances their therapeutic efficacy and enables the assessment of their potential off-target effects.

Another critical concern is the potential for secondary exosomes to be produced in response to the administered EVs. Such secondary exosomes could influence the therapeutic outcome or introduce unforeseen safety risks, emphasizing the need for comprehensive monitoring and characterization throughout the development of such EVs therapeutic agents.

Concerns about EVs from progenitor cells include the risk of carrying toxic or oncogenic cargo, triggering immune responses, and causing off-target effects in brain cells. Safety is ensured through rigorous EVs characterization, preclinical testing on brain models, and adherence to GMP for consistency and sterility. Continuous monitoring in clinical trials evaluates dose-response, immunogenicity, and long-term effects to mitigate risks.

Regarding their potential interactions with the immune system, EVs are considered therapeutics with low immunogenicity, especially when all EVs components are derived from the same species. However, EVs passage through fluids within the brain guarantees that at least some EV-based therapeutics will be taken up by the glymphatic system and transported to cervical lymph nodes. In light of the significant effects of naturally occurring EVs on lymph nodes in healthy individuals or in cancer patients (172, 173), the biological effects of new EVs-based therapeutics for brain conditions should be tested to ensure their safety.

Additionally, the long-term impact of EVs-based therapies on brain function and overall health must be carefully evaluated. Longitudinal studies of the EVs-based therapies are necessary to assess any potential adverse effects that may emerge over time, thereby ensuring their sustained safety and efficacy.

Altogether, prioritizing safety considerations of the EVs-based therapies at every stage of development can advance toward bringing safe and effective treatments to patients in need.

## 8 EVs-based therapies administered to human subjects

EVs-based therapies represent a novel approach that targets conditions not only in the brain but also in diseases anywhere in the human body. Given that the field is in its early stages of development, we are also reviewing clinical trials testing EV-based treatments in non-brain diseases. Published studies involving EV use in human subjects’ treatment include clinical trials at phase I or 2a.

### 8.1 EVs-based therapies in conditions that do not primarily affect the brain

The COVID-19 pandemic prompted several compassionate studies involving treatment with EVs derived from MSC in the bone marrow, adipose tissue, or umbilical cord (**Table 2**). In the study by Sengupta et al. (174, 175), 24 patients with severe COVID-19 disease received one intravenous dose of 15 ml EVs (corresponding to particles from 1-10 million cells/kg) (175), derived from a single donor-bone marrow MSC and co-administered with GM-CSF treatment. The authors reported that 71% of patients survived, 13% remained critically ill, and 16% died for reasons unrelated to the treatment. The patients’ clinical status and oxygenation improved; and their T lymphocyte counts increased, and acute phase reactants, such as C-reactive protein, ferritin, and D-dimers, significantly reduced. No adverse events were observed within 72 hours after the injection of EVs. Consistent with these findings, a 2a phase, single-arm, open-labeled clinical trial involving severe COVID-19 patients showed that nebulized EVs from allogeneic adipose tissue MSC (2x109 nanovesicles 1x per day for 5 days) was well tolerated. Furthermore, no pre-specified inhalation-associated adverse events were observed, suggesting a positive safety profile during and shortly after EV administration. All seven patients had a slight increase in blood lymphocytes, and four patients had different degrees of pulmonary lesion resolution (176). Similar effects were observed by Chu et al. with EVs derived from umbilical cord MSC (177). In this study, seven patients received adjuvant therapy of EVs nebulized in 5 ml saline containing 7.00-7.66*10^8^ particles/ml twice a day for 5-14 days, depending on each patient’s clinical status. The treatment was deemed safe and beneficial for the absorption of pulmonary lesions and hospital-day reduction.

**Table 2:** EV-based clinical trials addressing conditions unrelated to CNS.

Study/Condition	EVs Source	Delivery Route	Dosage	Outcomes	References
Severe COVID-19	Bone marrow MSC	Intravenous	15 ml (corresponding to particles from 1-10 million cells/kg)	71% survival, improved oxygenation, increased T lymphocytes, reduced CRP, ferritin, D-dimers, no adverse events.	(174, 175)
Severe COVID-19 (2a trial)	Adipose tissue MSC	Nebulized	2x10^9^ nanovesicles/day for 5 days	Well tolerated, no inhalation adverse events, increased lymphocytes, partial resolution of lung lesions.	(176)
Severe COVID-19	Umbilical cord MSC	Nebulized	5 ml (7.00-7.66x10^8^ particles/ml), twice/day	Safe, improved lung lesion absorption, reduced hospital stay.	(177)
Complex Perianal Fistula	Placenta MSC	Local application	0.5 × 10^10^ particles/mL that equals 50 μg/mL	Improved outcomes in 10/11 patients, safe.	(178)
Acne	Adipose tissue MSC	Gel application	9.78×1010 particles/ml	Significant improvement in outcomes.	(179)
Wound Healing	Allogeneic platelets	Topical application	100 *μ*g in 340 *μ*L	Safe, well tolerated in a double-blind Phase 1 trial.	(180)
Facial Skin Rejuvenation	Adipose tissue MSC	Microneedling (topical)		Improved skin outcomes in a 12-week randomized split-face study.	(181)
Radial Nerve Injury	Bone marrow MSC	Sub-epineural injection	1 ml (5x10^9^ particles/ml) in 4 doses	Satisfactory sensory and motor function recovery after 180 days.	(182)

MSC-derived EVs were also tested in human skin conditions, e.g., perianal fistula, acne, wound healing, and rejuvenation. For complex perianal fistula (persisting for more than a year despite medical and surgical treatment), EVs derived from placenta MSC was found to be safe and improved the outcomes in ten out of eleven patients (178). In acne, EVs derived from adipose tissue stem cells was administered in a gel as an adjuvant to a CO2 laser therapy and found to significantly improve outcomes (179). Concerning wound healing, a double-blind, placebo-controlled phase 1 trial on healthy volunteers established that the application of EV derived from allogeneic platelets is safe and well tolerated and can now be studied in further trials (180). Concerning rejuvenation, application of EV-derived human adipose tissue stem cells improved the outcome of micro-needling for facial skin in a 12-week prospective, randomized split-face study (181).

More recently, a case report of a 24-year-old male with total radial nerve injury was published (182). The patient was treated surgically and received a sub-epineural injection of MSC-derived EVs (1 ml of 5x10^9^ particles/ml divided into four doses applied on one occasion at different parts of the injured nerve). The patient was followed for 180 days, and the patient’s sensory and motor function recovery was satisfactory.

Altogether, the studies discussed above and outlined in the **Table 2** suggest that MSC-derived EVs derived from various tissue origins promote healing and is safe. Nonetheless, standardization of EVs generation, testing their dose- responses, and monitoring for possible adverse effects over longer periods is still needed.

### 8.2 EVs-based therapies in conditions affecting the brain

The two most common diseases that affect the brain are neurological conditions, namely stroke and Alzheimer’s disease (**Table 3**). The possibility of treating these conditions with MSC-derived EVs is supported by clinical trials assessing the safety of the intervention. Concerning stroke, an open-label randomized clinical trial tested the impact of intraparenchymal injection of placenta-derived MSC-derived EVs performed during decompression craniotomy in five male patients (aged 56-70 years) who suffered brain infarction due to malignant middle cerebral artery occlusion leading to brain infarction (183). No serious adverse effects at the single dose of 356 µg/ml related to the injection of EVs were observed during a 3-month follow-up visit. This study was the first to test the EVs administration’s clinical feasibility and safety when introduced to the brain.

**Table 3:** EVs-based therapies for CNS conditions.

Condition	EVs Source	Delivery Route	Dosage	Outcomes	References
Stroke	Placenta-derived MSC	Intraparenchymal injection	Single dose of 356 µg/ml	No serious adverse effects, safety confirmed during 3-month follow-up.	(101)
Alzheimer’s Disease	Human adipose MSC	Intranasal	4×10^8^ particles/ml, twice/week for 12 weeks	Improved cognitive function; no change in amyloid plaques or tau deposition.	(184)
Refractory Focal Epilepsy	Induced pluripotent stem cells (iPSC)	Nasal drops	2-18 µg in 200µl, 2x/day, 12 weeks	Ongoing study; investigating efficacy for epilepsy treatment.	NCT05886205
Acute Ischemic Stroke	iPSC-derived EV	Intravenous	2×10^9 particles/kg	Ongoing study to evaluate safety and efficacy.	NCT06138210
Extremely Low Birth Weight Infants	MSC-derived EV	Intravenous	1 dose – from 120 million MSC	Pilot experiment investigating neuroprotective effects.	NCT05490173

Concerning treatment of Alzheimer’s disease with EVs, in an open-label clinical trial I-II, patients received intranasal EVs derived from human adipose MSC at the dose of 4×10^8^ particles/ml twice per week for 12 weeks. Then they were checked during the follow-up visits at weeks 16, 24, 36 and 48 (184). The treatment improved their cognitive functions, though their amyloid plaques or tau deposition remained the same.

Additional ongoing efforts in the field that have not yet been published can be found in the comprehensive database of publicly and privately funded clinical trials, *Clinicaltrials.gov*. As of Dec 31, 2024, EVs were investigated in 360 clinical studies, of which seventy-one focused on treating various conditions. Only a handful of interventional studies are devoted to neurological or neuropsychiatric conditions. They include a trial on nasal drops with EVs from induced pluripotent stem cells (iPSC) for refractory focal epilepsy treatment (NCT05886205), a study of safety and efficacy of intravenous iPSC-derived EVs for acute ischemic stroke (NCT06138210) and a pilot experiment of neuroprotective effects of MSC-derived EVs (not specified further) in extremely low -weight infants (NCT 05490173) (**Table 3**).

These initial reports and ongoing efforts are clinical trials at phase I or I-II. Limitations of these interventional studies include the absence of control subjects or groups treated with a placebo. Also, EVs employed in these studies are derived from different MSC sources, and no information about the cargo of the therapeutic EVs that could allow for standardization of the sources. Thus far, no clinical trials with artificial or hybrid EVs have been reported.

## 9 Limitations

While EVs-based therapies hold promise for treating neuropsychiatric conditions, several limitations and challenges need to be addressed in interdisciplinary translational research, hopefully in the immediate or near future.

First, the diagnosis of psychiatric conditions currently relies primarily on the neuro-behavioral system, but significant efforts are devoted identifying biochemical, immunological, and imaging-based biomarkers. The dynamic interactions among neuronal, glial, immune, and EV networks are still poorly understood. Their complexity complicates the identification of specific therapeutic targets and biomarkers. The resolution may come from designing extensive, multisite studies that can lead to the identification of subsets of patients with different pathogenic mechanisms. An example of such efforts is the SFARI (Simon’s Foundation for Autism Research Initiative; https://gene.sfari.org/). At the beginning of its extensive, multisite efforts, the same and uniformly-applied neuro-behavioral diagnostic approach was adopted at all sites in recruiting and evaluating patients. The investigators created a large biobank that enabled both systematic addressing of well-designed pertinent questions, as well as open availability of patient samples for individual investigators to test novel ideas.

Second, the lack of standardized protocols for EV isolation, characterization, and cargo profiling remains a significant hurdle (185). Without standardized protocols, reproducibility across studies and clinical applications is challenging. Close collaboration with experts in the field will be needed to assure the selection of the optimal approaches and then the consistent application of the optimal approach throughout such a study of a large number of individuals can ultimately lead to the generation of comparable data for patients with a given psychiatric disease for evaluation.

Third, while brain fluid dynamics are recognized as critical to the four interacting networks (namely, the neuronal, glial, the body’s immune system, and the EVs), the mechanisms by which abnormalities (e.g., edema, CSF alterations) affect EVs function and therapy remain largely unexplored (186). Collaborative translational efforts among clinical and experimental investigators in these fields will be needed to facilitate the closing of the gaps in our knowledge of these important and clinically relevant areas.

Fourth, regulatory requirements for EVs-based therapies are still evolving. Safety, efficacy, and consistency will require substantial preclinical and clinical data.

Fifth, the development of EVs-based therapies requires close collaborative research across multiple disciplines. Such collaborative efforts can be resource-intensive and complex and need to involve both academic and non-academic sectors, as exemplified by the development of adaptive clinical trials in breast cancer research (187).

## 10 Conclusions and future directions

EVs-based therapies offer exciting potential for new ways of diagnosing and treating psychiatric conditions by practicing personalized and precise medicine, which may ultimately lead to a decrease in the number of patients suffering resistance to existing neuropsychiatric medications.

Facing the complexity of brain structure and functions and investigating neuronal, glial, immune, and EVs networks together may open new avenues for discovering new and objective biomarkers.

EVs-based interventions in immune and/or inflammatory processes in the brain may become a significant tool for understanding how important immune factors play in the pathogenesis of brain diseases. Targeting approaches to deliver EVs to specific cells within the brain will also likely remove the concerns about iatrogenic deficiency.

Challenges of manufacturing EVs-base therapies include standardizing sources and production of EVs on a larger scale.

A critical aspect of clinical research is the lack of in-depth knowledge about physiological processes within the brain and understanding how it can adapt to various stimuli without causing irreversible disrepair. Understanding these fundamental aspects and how homeostasis changes in chronic disease will undoubtedly facilitate improvements in diagnoses and treatments of psychiatric conditions. EVs with their regulatory properties and focused cellular impact can be employed in *in vitro* 2D or 3D cultures with human cells *or in vivo* experimental animals represent a great tool to improve our knowledge of these fundamental processes.

Progress in the development of EVs-based therapeutics will likely come from integrated, interdisciplinary collaborative efforts on large preclinical and clinical studies. Such translational efforts need to focus on elucidating the mechanisms underlying therapeutic effects and the possibility of identifying markers that allow targeting specific cell types.

As interdisciplinary research progresses and new data emerge, regulatory agencies are expected to demand more comprehensive information to ensure the highest possible safety standards for novel therapeutics.
